# Determinants of Intention to Participate in Breast Cancer Screening among Urban Chinese Women: An Application of the Protection Motivation Theory

**DOI:** 10.3390/ijerph182111093

**Published:** 2021-10-21

**Authors:** Miao Zhang, Wenshuang Wei, Qinmei Li, Xinguang Chen, Min Zhang, Dan Zuo, Qing Liu

**Affiliations:** 1 Department of Epidemiology, School of Health Sciences, Wuhan University, Wuhan 430071, China; zhangmiao@whu.edu.cn (M.Z.); 2020283050097@whu.edu.cn (W.W.); 00300654@whu.edu.cn (D.Z.); 2 Wuhan Center for Disease Control and Prevention, Wuhan 430015, China; qinmei.li@whu.edu.cn; 3 Department of Epidemiology, University of Florida, Gainesville, FL 32610, USA; jimax.chen@ufl.edu; 4 Institute of Cancer Prevention and Control, Wuhan 430079, China; hbskaxh@163.com

**Keywords:** breast cancer, screening, China, urban women, protection motivation theory (PMT), structural equation modeling (SEM)

## Abstract

Despite the significance of early detection of breast cancer through screening, the screening uptake in China remains relatively low. Protection motivation theory (PMT) suggested by Rogers is one of the theories concerning threat appeal. This study aimed to apply the protection motivation theory (PMT) in predicting breast cancer screening intention. In this cross-sectional study, a sample of Chinese urban women was recruited using the convenient sampling method from five communities in Wuhan. Data were collected using a self-report questionnaire that included demographic variables, knowledge about breast cancer, six PMT subconstructs, and screening intention. We used the structural equation modeling (SEM) to identify the predictor factors associated with screening intention. Of the total sample (*n* = 412), 86.65% had intention to participate in screening. Our data fit the hypothesized SEM model well (Goodness of fit index (GFI) = 0.91, adjusted GFI (AGFI) = 0.89, comparative fit index (CFI) = 0.91, root mean square error of approximation (RMSEA) = 0.05, standardized root mean residual (SRMR) = 0.06, and Chi-square/df = 2.01). Three PMT subconstructs (perceived severity, response cost, and self-efficacy) were significantly associated with screening intention. Knowledge, social status, and medical history had significantly indirect associations with screening intention through the mediating effect of PMT subconstructs. Considering the utility of PMT, intervention programs might be more effective based on the subconstructs of PMT, especially to improve self-efficacy, perceived severity, and knowledge, reduce response cost, as well as targeting specific demographic groups.

## 1. Introduction

Breast cancer is the most common cancer and the leading cause of cancer-related death in women worldwide. According to the latest data released by the International Agency for Research on Cancer (IARC), breast cancer has surpassed lung cancer as the most common cancer globally, with 2.3 million new cases in 2020 [1]. More than half of these breast cancer cases occurred in low- and middle-income countries. In China, there were 420,000 new cases of breast cancer and 120,000 deaths in 2020. Additionally, breast cancer incidence among Chinese women has increased more than twice as fast as the global rates, particularly in urban areas [2]. Obviously, it poses a serious threat to public health and portends an ever-increasing burden of breast cancer in China.

The early detection of breast cancer could significantly reduce mortality and the disease burden [3,4]. Mammography is the most effective screening method since it helps in early diagnosis and treatment in the asymptomatic stage [5]. Despite vast evidence regarding the importance of regular mammography [3,4,5,6], the screening participation rates remain low in China and vary greatly by age, region, and insurance status [7,8]. The Chinese government has carried out the population-based Cancer Screening Program in Urban China (CanSPUC) since 2012, which includes free biennial breast cancer screening for all women aged 40–74 years [9]. In China, the average costs of breast cancer screening were 291,210 (45,505.83 USD) and 886,050 yuan (138,458.29 USD) per cancer case detected in urban and rural areas, respectively [10]. A large-scale population-based study among Chinese women demonstrated that only 22.5% of 35–69-year-olds had ever undergone breast cancer screening [11]. A lack of knowledge and unawareness of the necessity of screening is a significant barrier to widespread participation in mammography screening [12,13]. Other factors that affect the screening uptake include education level, occupation, a personal history of breast disease, cost and time constraint, fear, and embarrassment [14,15,16,17,18,19,20].

Health behavior theory can help to explain and alter the disproportionately low rates of screening [21]. The health belief model (HBM) is the most common theory for promoting breast cancer screening behavior [22]. This model is one of the most widely used social cognition models to predict health behaviors and is founded on six fundamental constructs: perceived susceptibility, perceived severity, perceived benefits, perceived barriers, cues to action, and self-efficacy [23]. Previous research has suggested that HBM-based educational interventions could help to improve knowledge and beliefs about breast cancer and, subsequently, to promote screening uptake among women [22,24]. However, this model has limitations in that it places narrowly defined determinants of behavior in a specific relationship to one another, completely isolated from social context [21]. Moreover, associations between cancer screening behavior and HBM constructs have not been identified in some studies [25,26].

Protection motivation theory (PMT) was proposed by Rogers based on HBM in 1975 [27]. It is a cognitive social theory used to predict various health behaviors, such as schistosomiasis prevention [28], vaccination behavior [29], and cancer screening [30,31,32]. PMT considers threat appraisal and coping appraisal as the main predictors of health behavior [27]. Specifically, individuals’ protection motivation would increase as the levels of threat and coping appraisal increase, which would increase the likelihood of performing a health behavior. According to PMT, intention is the most proximal predictor of behavior. In turn, intention is determined by two parallel cognitive processes: threat appraisal and coping appraisal. Threat appraisal evaluates a person’s perception of the threat of diseases or certain behaviors using the three subconstructs of perceived risk, perceived severity, and fear arousal. Meanwhile, coping appraisal assesses an individual’s ability to respond to and avert the threatened danger using the three subconstructs of response efficacy, response cost, and self-efficacy.

Numerous studies have been conducted to investigate the factors that may affect women’s participation in breast screening [17,33,34], but there remains relatively limited research on this topic in China, especially studies based on PMT. Previous studies in Iran have identified the importance of several PMT subcontracts in predicting breast cancer screening [35,36,37]. In these studies, increased self-efficacy, response efficacy, and perceived severity were found to be significantly associated with breast cancer screening behavior. However, China has a different cultural background and screening services, so findings from other countries might not be applicable in China. Therefore, exploring the intention of breast cancer screening and its associated factors in China is urgently needed.

This study aimed to determine the predictor factors associated with breast cancer screening intention among urban Chinese women, and identify the associations of PMT subconstructs with screening intention. The goal of our study was to provide an evidence base for designing intervention strategies for breast cancer screening in the future.

## 2. Materials and Methods

### 2.1. Design

A cross-sectional survey was conducted in 2020. This study is part of the Cancer Screening Program in Urban China (CanSPUC). This project was organized by the National Health and Family Planning Commission. Since 2012, screening programs for the five major types of cancer (lung cancer, colorectal cancer, upper gastrointestinal cancer, breast cancer, and liver cancer) have been carried out for risk factor investigation, high-risk population assessment, cancer screening, and health economics assessment. Our study was a breast cancer screening intention survey when screening high-risk groups at baseline. The high-risk population was identified by the Harvard Cancer Risk Index to calculate an individual risk score for breast cancer exposure, which included history of benign breast disease, family history of breast cancer, age at menarche, childbirth history, reproductive age, and breastfeeding history [38]. The relative risk score was calculated by comparing the individual risk score with the overall national average [11]. The high-risk group for breast cancer was defined as individuals with a relative risk > 2.

### 2.2. Participants

This study was conducted in Wuhan, the capital city of Hubei Province, an important central city in mainland China. Participants were selected using the convenient sampling method from five communities. All eligible participants who resided in the sampled communities were invited to participate in the study through WeChat (version 8.0.11) by staff of the community neighborhood committees. The inclusion criteria were as follows: (a) Aged 40–74 years and (b) willing to participate. Women with a history of breast cancer or suspected breast cancer were excluded. The study focused only on women aged 40–74 years, because epidemiological evidence demonstrated that the peak onset age for breast cancer in Chinese women is between 40 and 50 years, and women with an average risk of breast cancer should begin mammography at 40 years old [39]. A total of 435 questionnaires were distributed; 412 (94.7%) valid questionnaires were returned. Data from 23 participants were excluded, because 20 had missing demographic data and three had missing PMT data, yielding a final sample of 412.

### 2.3. Data Collection

Data were collected in September and October, 2020. The survey was administered by trained graduate students from Wuhan University. After consent was obtained, data were collected through person-to-person interviews in the community health service center settings using pencil and paper questionnaires that had been pilot-tested, which took approximately 15 min. To minimize bias, all investigators received two-day professional training before distributing the surveys. The completed questionnaires were checked, verified, and recalled by the trained data collectors on the spot.

### 2.4. Measures

#### 2.4.1. Demographic Variables

The demographic variables included age (in years), education (primary or less, junior high school, senior high/technical school, and junior college/college and above), marital status (single, married, and widowed/divorced), occupation (employees of enterprises and institutions, industrial/commercial/services workers, farmer, housewife, and unemployed/retired), history of benign breast disease (yes/no), and family history of breast cancer (yes/no). For the subsequent SEM analysis, education and occupation were merged into a latent variable called “social status”. Education was categorized into four scores (primary or less = 1, junior high school = 2, senior high/technical school = 3, and junior college/college and above = 4). Occupation was categorized into five scores (unemployed/retired = 1, housewife = 2, farmer = 3, industrial/commercial/services workers = 4, and employees of enterprises and institutions = 5). The total score of “social status” was obtained by adding up the score of education and occupation, which ranged from 2 to 9. Similarly, history of benign breast disease and family history of breast cancer were merged into a latent variable called “medical history”. Each item was rated as “yes” (1 point) or “no” (0 point) and the points were combined to obtain a total score of “medical history” ranging from 0 to 2.

#### 2.4.2. Knowledge of Breast Cancer

The knowledge of breast cancer scale consists of 3 subscales. Knowledge of risk factors included seven items and was assessed by asking women to select “yes” or “no” to whether the items could increase the risk of breast cancer. Knowledge of symptoms included five items and was assessed by asking women whether the items were breast cancer signs. Knowledge of breast cancer screening included three items and assessed whether women were aware of mammography screening. For knowledge of breast cancer, each correct answer was given one point, with a maximum of 15 points.

#### 2.4.3. Breast Cancer PMT Scale

The Breast Cancer PMT Scale was developed by the authors based on a literature review and expert consultation. The reliability and validity of the scale were confirmed among Chinese women [40]. The items of the Breast Cancer PMT Scale are presented in Table A1. The Cronbach’s α coefficient was 0.80 for the PMT instrument. Exploratory factor analysis showed that the rotated factor loads of the items varied from 0.51 to 0.88, and the 17 factors explained 68.39% of the observed variance. The correlation of each PMT subconstruct with the total scale varied between 0.36 and 0.77. The Breast Cancer PMT scale includes six subconstructs: (1) Perceived risk referred to an individual’s subjective judgement of the possibility of getting breast cancer (e.g., I am more likely to get breast cancer compared with others), which consists of two items (Cronbach’s α = 0.37). (2) Perceived severity referred to personal subjective judgement of negative consequences from breast cancer (e.g., getting breast cancer will seriously affect my health), which consists of three items (Cronbach’s α = 0.82). (3) Fear arousal referred to an individual’s worry or concern about being affected by breast cancer (e.g., I will worry about getting breast cancer), which consists of two items (Cronbach’s α = 0.66). (4) Response efficacy referred to an individual’s judgement of the effectiveness of the early detection and treatment of breast cancer (e.g., only by doing mammography, can breast cancer be detected early), which consists of three items (Cronbach’s α = 0.79). (5) Response cost referred to an individual’s subjective assessment of the costs associated with participating in breast cancer screening (e.g., I feel embarrassed to receive mammography), which consists of three items (Cronbach’s α = 0.78). (6) Self-efficacy referred to individuals’ belief in their capabilities and confidence to engage in breast cancer screening (e.g., I have enough time to do mammography examination), which consists of four items (Cronbach’s α = 0.74).

The 17 items of the Breast Cancer PMT Scale were measured on a five-point Likert scale ranging from 1 (strongly disagree) to 5 (strongly agree). The mean score of each PMT subconstruct was computed from the individual items, and higher score represents a greater perception about that subconstruct.

#### 2.4.4. Intention to Participate in Breast Cancer Screening

One item was used to assess screening intention by asking “Are you willing to participate in breast cancer screening in the future?” using a five-point scale ranging from 1 (very unwilling) to 5 (very willing). For our analyses, we integrated screening intention scores of “4” and “5” into “Yes”, which represented intention to participate in breast cancer screening; meanwhile, scores “1” to “3” were integrated into “No”, indicating no screening intention.

### 2.5. Statistical Analysis

Data were double entered in EpiData (version 3.2) after checking them manually. Errors identified by comparing the double data entries were resolved by referring to the paper records. We used descriptive statistics to summarize the sociodemographic characteristics of the study sample. Comparisons between groups were determined using the Chi-square or *t*-tests. Pearson’s correlation was used to assess the relationship between the PMT constructs and screening intention. The structural equation model (SEM) was employed to assess the relationship between variables, and data model fitting was assessed using the following indexes: Goodness of fit index (GFI) > 0.9, adjusted GFI (AGFI) > 0.9, comparative fit index (CFI) > 0.9, root mean square error of approximation (RMSEA) < 0.08, standardized root mean residual (SRMR) < 0.05, and Chi-square/df < 5.0. The statistical significance level was set at *p* < 0.05. IBM SPSS statistics for windows (version 25.0) and Amos (version 21.0) were used for all analyses.

## 3. Results

### 3.1. Sample Characteristics

The sociodemographic characteristics of the study sample are presented in Table 1. Of the total sample (*n* = 412), 86.65% expressed the willingness to screen for breast cancer in the future. More than half (52.18%) of the participants were over 60 years old, and the majority (94.17%) were married. Approximately half (50.48%) of the participants had less than a senior high school education, and 47.57% were industrial/commercial/services workers. Of the whole sample, 65.53% had no personal history of benign breast disease, and 95.15% had no family history of breast cancer.

### 3.2. Knowledge of Breast Cancer

Table 2 presents the mean score for each item of the breast cancer knowledge scale among participants with and without intention. The results of the *t*-tests showed that the knowledge score of breast cancer was significantly higher among those women with screening intention than those without (*p* < 0.05).

### 3.3. Association of the PMT Subconstructs with Screening Intention

As shown in Table 3, the Pearson’s correlation analysis indicated that screening intention was significantly associated with perceived severity (*r* = 0.27, *p* < 0.01), fear arousal (*r* = 0.21, *p* < 0.01), response efficacy (*r* = 0.22, *p* < 0.01), response cost (*r* = –0.31, *p* < 0.01), and self-efficacy (*r* = 0.47, *p* < 0.01).

### 3.4. Structural Equation Modeling

**Figure 1:**
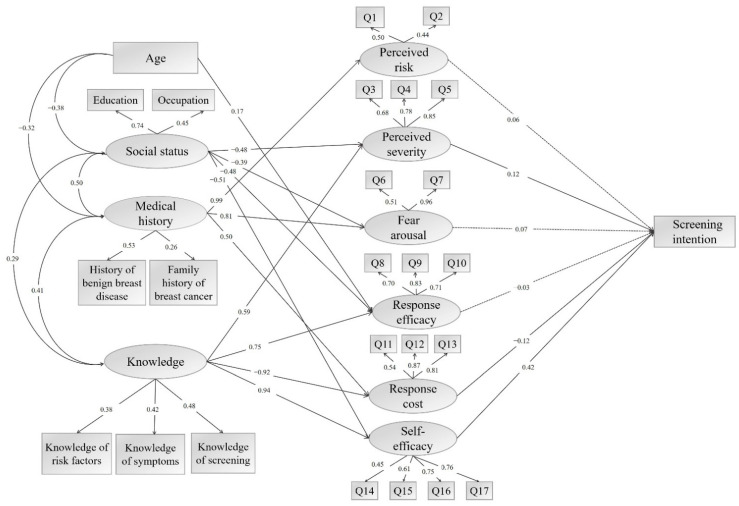
Results from PMT-guided SEM analysis of the factors related to screening intention. Note: Data-model fit index: GFI = 0.91, AGFI = 0.89, CFI = 0.91, RMSEA = 0.05, SRMR = 0.06, and Chi-square/df = 2.01. The latent variables are represented by ovals, while the observed variables are represented by rectangles. The observed variables “Q1–Q17” represent the 17 items of the Breast Cancer PMT Scale (see Table A1). The values of the single-headed arrows indicate the standardized coefficients. A solid line indicates a statistically significant association with *p* < 0.05 and a dashed line indicates the association was not statistically significant.

Figure 1 presents the results from the structural equation modeling. The fit indices show that the hypothesized model fit the data acceptably well (GFI = 0.91, AGFI = 0.89, CFI = 0.91, RMSEA = 0.05, SRMR = 0.06, and Chi-square/df = 2.01). First, among the six PMT subconstructs, perceived severity (coefficient = 0.12, *p* < 0.05), response cost (coefficient = –0.12, *p* < 0.05), and self-efficacy (coefficient = 0.42, *p* < 0.05) were significantly associated with breast cancer screening intention. Perceived severity measures the perceived negative consequences from breast cancer, response cost measures the perceived costs associated with breast cancer screening, and self-efficacy measures the perceived ability to take part in breast cancer screening.

Of the four external factors, knowledge of breast cancer was indirectly associated with screening intention mediated by three significant PMT subconstructs. Knowledge was significantly positively associated with perceived severity (coefficient = 0.59, *p* < 0.05) and self-efficacy (coefficient = 0.94, *p* < 0.05), while it was significantly negatively associated with response cost (coefficient = –0.92, *p* < 0.05). In other words, knowledge can promote screening intention by increasing the perceived severity and self-efficacy, as well as reducing response cost.

Lastly, social status and medical history had indirect associations with screening intention mediated through different PMT subconstructs. Social status was significantly associated with perceived severity (coefficient = –0.48, *p* < 0.05) and self-efficacy (coefficient = –0.51, *p* < 0.05), while medical history was significantly associated with response cost (coefficient = 0.50, *p* < 0.05).

## 4. Discussion

In this study, we examined both the direct and indirect effects of the predictive factors on women’s intention of breast cancer screening guided by PMT. The results from the SEM analysis proved the validity of PMT for predicting screening intention. This study provides an evidence base for designing intervention strategies and improving the screening uptake in China.

The results from the path analysis showed that self-efficacy, perceived severity, and response cost were significant PMT constructs determining women’s screening intention. As several PMT meta-analyses have indicated, the specificity of the PMT measurement for special populations are crucial, especially when PMT-based studies are used to guide health policy at an operational level [41,42]. A meta-analysis found that among all PMT variables, the strengths of predicting health behavior or intention were not the same [41]. Moreover, in our study, the influence of the PMT subconstructs on screening intention showed different strengths, which should be a focus in future intervention programs.

The findings of this study showed that self-efficacy was positively associated with screening intention. Similar to our results, in other studies, women with higher self-efficacy were more likely to perform screening regularly [36,37,43,44]. A meta-analysis of the PMT literature also identified that self-efficacy plays the strongest role in predicting intention [41]. This calls for more attention on self-efficacy in intervention programs to increase women’s screening attendance. On the one hand, women’s beliefs in their capabilities to engage in screening should be improved through conveying messages to address the health benefits of mammography in earlier-stage detection. On the other hand, the presentation of successful examples and the encouragement of those who have regular screening behaviors could be an effective strategy to enhance women’s perceived ability to engage in screening.

Another PMT predictor of women’s intention to undergo screening was perceived severity, which was in accordance with previous studies [32,45]. A quasi-experimental study in Iran suggested that women are more likely to perform the screenings if they understand the severity and harm of the disease as well as the related consequences [46]. When individuals have low perceived severity about breast cancer, they have a low motivation to perform the recommended preventive measures [37]. For this reason, information about breast cancer threat should be emphasized among women by healthcare providers and community advocators to increase their awareness of the severity of the disease.

In this study, we found that there was a negative association of the response cost with breast screening intention, which indicates that the perceived costs of mammography could be a barrier for women to engage in screening. A qualitative study based on PMT found that embarrassment associated with mammography was considered as a vital response cost [47]. Chinese women believed that mammography was time-consuming [17], and fear of embarrassment when exposing their breasts to someone else discouraged them from performing mammography [18]. Effective measures should be taken to reduce women’s perceived costs for screening, including protecting their privacy, giving appropriate advice regarding breast health practices to reduce their negative feelings, and improving the efficiency of mammograms to reduce the waiting time.

Effective educational programs should also emphasize the significance of knowledge of breast cancer. Our findings suggest that enhanced knowledge could help improve PMT subconstructs, and subsequently increase women’s intention to undergo mammography screening. Meanwhile, limited knowledge regarding breast cancer was observed in our study, which is consistent with other studies [14,48,49,50]. It is not surprising that the public have low awareness of breast cancer, as the issue of cancer is often considered taboo in Chinese culture [51]. Given this situation, future health education should emphasize increasing knowledge about breast cancer in terms of risk factors, symptoms, and the necessity of mammography screening. These may help eliminate women’s misunderstandings about breast cancer and strengthen their awareness of regular screening.

This study also demonstrated the role of other factors in screening intention. The results from our study found that medical history (history of benign breast disease and family history of breast cancer) and social status (education and occupation) were associated with various PMT subconstructs. For example, medical history was indirectly associated with screening intention mediated through response cost, so there was a lower probability of mammography among women with a medical history. One possible explanation is that fatalism is more common in women with a medical history [52]. Previous studies have indicated that women with a family history of breast cancer or with a history of benign breast disease, who are more likely to believe in fatalism, may consider mammography an ineffective and high-cost screening method [52,53,54]. This may also explain the positive association between medical history and response cost, suggesting that in addition to PMT subconstructs, breast cancer screening programs might also benefit by considering these socio-demographic factors.

As breast cancer remains an increasingly serious public health issue in China, understanding women’s screening intention and behavior is the key step to form tailored breast cancer screening and prevention strategies. The present study confirmed the important role of PMT in cancer screening interventions, which may be useful for healthcare workers to implement theory-guided strategies to increase the possibility of taking up screening. Importantly, we identified PMT constructs in the promotion of screening uptake, such as self-efficacy, perceived severity, and response cost, which should be considered targets for future intervention of breast cancer screening. More attention is also needed for women with a family history of breast cancer and history of benign breast disease. Moreover, popularizing knowledge about breast cancer is another strategy to promote screening participation for women.

This study has several limitations. First, it was a cross-sectional design, so no causal relationships could be inferred, and longitudinal studies are required to further confirm our findings. Second, this study only included respondents from Wuhan, so the results cannot be generalized to other regions in China, a country with more than 1.4 billion citizens with diverse socioeconomic and cultural backgrounds. Future research should include larger samples and replicate and expand this work in urban settings. Lastly, although the reliability of the total PMT scale was acceptable, the Cronbach’s α coefficients for two PMT subconstructs were less than satisfactory: perceived risk (Cronbach’s α = 0.37) and fear arousal (Cronbach’s α = 0.66). Thus, the reliability of these two subconstructs should be improved in future research.

## 5. Conclusions

Despite these limitations, this study examined the PMT’s predictive validity for breast cancer screening intention among Chinese urban women. It identified that self-efficacy, response cost, perceived severity, and knowledge as well as some demographic factors were significant predictors of breast cancer screening intentions. The findings of this study provide new data supporting more effective interventions to increase the uptake of breast screening.

## Figures and Tables

**Table 1:** Characteristics of the study sample.

Variables	Screening Intention		Total (%)
	Yes	No	
Sample size, *N* (%)	357 (86.65)	55 (13.35)	412 (100.00)
Age in years, *n* (%)			
40–49	57 (93.44)	4 (6.56)	61 (100.00)
50–59	118 (86.76)	18 (13.24)	136 (100.00)
60–74	182 (84.65)	33 (15.35)	215 (100.00)
Marital status, *n* (%)			
Single	3 (100.00)	0 (0.00)	3 (100.00)
Married	336 (86.60)	52 (13.40)	388 (100.00)
Widowed/divorced	18 (85.71)	3 (14.29)	21 (100.00)
Education, *n* (%)			
Primary or less	81 (84.38)	15 (15.63)	96 (100.00)
Junior high school	93 (83.04)	19 (16.96)	112 (100.00)
Senior high/technical school	106 (90.60)	11 (9.40)	117 (100.00)
Junior college/college and above	77 (88.51)	10 (2.43)	87(100.00)
Occupation, *n* (%)			
Employees of enterprises and institutions	102 (89.47)	12 (10.53)	114 (100.00)
Industrial/commercial/services workers	170 (86.73)	26 (13.27)	196 (100.00)
Farmer	54 (85.71)	9 (14.29)	63 (100.00)
Housewife	18 (81.82)	4 (18.18)	22 (100.00)
Unemployed/retired	13 (76.47)	4 (23.53)	17 (100.00)
History of benign breast disease, *n* (%)			
Yes	125 (88.03)	17 (11.97)	142 (100.00)
No	232 (85.93)	38 (14.07)	270 (100.00)
Family history of breast cancer, *n* (%)			
Yes	18 (90.00)	2 (10.00)	20 (100.00)
No	339 (86.48)	53 (13.52)	392 (100.00)

**Table 2:** Comparison of knowledge about breast cancer among urban women with and without screening intention in Wuhan, China.

Total Scale/Single Item	Screening Intention		*p*-Value
	Yes	No	
Total scale score, mean (SD)	6.80 (3.61)	5.07 (3.66)	0.001 **
Knowledge of risk factors for breast cancer			
subscale score, mean (SD)	2.80 (1.95)	2.18 (2.13)	0.030 *
Older age, *n* (%)	171 (47.90)	17 (30.91)	0.019 *
Obesity, *n* (%)	142 (39.78)	16 (29.09)	0.129
Oral contraceptive use, *n* (%)	131 (36.69)	14 (25.45)	0.104
Non-breastfeeding, *n* (%)	213 (59.66)	27 (49.09)	0.139
Family history of breast cancer, *n* (%)	190 (53.22)	24 (43.64)	0.185
Menarche at age before 13, *n* (%)	71 (19.89)	10 (18.18)	0.767
Menopause at age over 55, *n* (%)	83 (23.25)	12 (21.82)	0.815
Knowledge of symptoms for breast cancer			
subscale score, mean (SD)	3.39 (1.69)	2.65 (1.82)	0.003 **
Lump in breast, *n* (%)	293 (82.07)	39 (70.91)	0.051
Change in breast texture, *n* (%)	189 (52.94)	24 (43.64)	0.199
Axillary mass, *n* (%)	259 (72.55)	34 (61.82)	0.102
Nipple turned inward into the breast, *n* (%)	221 (91.32)	21 (38.18)	0.001 **
Discharge from nipple, *n* (%)	247 (69.19)	28 (50.91)	0.007 **
Knowledge of breast cancer screening			
subscale score, mean (SD)	0.61 (1.10)	0.24 (0.64)	0.000 **
Mammography is one of the methods of breast cancer screening, *n* (%)	83 (23.25)	8 (14.55)	0.147
Mammography is recommended every year among women over 40 years old, *n* (%)	51 (14.29)	1 (1.82)	0.010 *
Breast cancer can be detected early by mammography screening, *n* (%)	85 (23.81)	4 (7.27)	0.006 **

Note: SD, standard deviation. ** *p* < 0.01 and * *p* < 0.05.

**Table 3:** Correlations of breast cancer screening intention with PMT subconstructs.

Variables	X2	X3	X4	X5	X6	X7
X1 Screening intention	0.09	0.27 **	0.21 **	0.22 **	–0.31 **	0.47 **
X2 Perceived risk		–0.01	0.36 **	0.03	0.05	0.15 **
X3 Perceived severity			0.22 **	0.29 **	–0.25 **	0.26 **
X4 Fear arousal				0.11 *	0.03	0.16 **
X5 Response efficacy					–0.30 **	0.38 **
X6 Response cost						–0.48 **
X7 Self-efficacy						1.00

Note: ** *p* < 0.01 and * *p* < 0.05.

## Data Availability

The data presented in this study are openly available contacting corresponding Author.

## Appendix A

**Table A1:** Items of the 17-Item Breast Cancer PMT Scale.

Item by Subconstructs	Mean (SD)
Perceived risk	5.19 (1.71)
Q1. I am more likely to get breast cancer compared with others.	2.44 (0.94)
Q2. Someone once reminded me to be careful of getting breast cancer.	2.75 (1.23)
Perceived severity	13.26 (1.90)
Q3. Getting breast cancer will seriously affect my health.	4.48 (0.71)
Q4. Once I have breast cancer, my life will change significantly.	4.33 (0.81)
Q5. The impact of breast cancer on my whole family is huge.	4.45 (0.70)
Fear arousal	6.81 (2.18)
Q6. When I think of breast cancer, I get nervous.	3.73 (1.21)
Q7. I will worry about getting breast cancer.	3.08 (1.32)
Response efficacy	11.44 (2.28)
Q8. Only by doing mammography, can breast cancer be detected early.	3.77 (0.97)
Q9. Through mammography examination, I can figure out whether I have breast cancer.	3.94 (0.81)
Q10. If someone wants to cure breast cancer, she can’t do it without mammography.	3.73 (0.94)
Response cost	6.00 (2.43)
Q11. I feel embarrassed to receive mammography.	4.04 (0.98)
Q12. Doing mammography examination is too time-consuming.	4.00 (1.00)
Q13. Doing mammography examination is too wasteful of money.	3.93 (0.99)
Self-efficacy	15.08 (2.98)
Q14. I have enough time to do mammography examination.	3.68 (1.08)
Q15. Mammography is very easy for me to accept.	3.95 (0.87)
Q16. Even if others say that mammography is not necessary, I will do it myself.	3.69 (1.01)
Q17. Even if I have to pay for it, I will go for mammography.	3.76 (1.02)
